# Draft Genome Sequence of Gerbil-Adapted Carcinogenic *Helicobacter pylori* Strain 7.13

**DOI:** 10.1128/genomeA.00641-15

**Published:** 2015-06-11

**Authors:** Mohammad Asim, Surendra K. Chikara, Arpita Ghosh, Srinivas Vudathala, Judith Romero-Gallo, Uma S. Krishna, Keith T. Wilson, Dawn A. Israel, Richard M. Peek, Rupesh Chaturvedi

**Affiliations:** a Division of Gastroenterology, Hepatology, and Nutrition, Department of Medicine, Vanderbilt University Medical Center, Nashville, Tennessee, USA; b Department of Cancer Biology, Vanderbilt University Medical Center, Nashville, Tennessee, USA; c Veterans Affairs Tennessee Valley Healthcare System, Nashville, Tennessee, USA; d Xcelris Labs Ltd., Ahmedabad, India; e School of Biotechnology, Jawaharlal Nehru University, New Delhi, India

## Abstract

We report here the draft genome sequence of *Helicobacter pylori* strain 7.13, a gerbil-adapted strain that causes gastric cancer in gerbils. Strain 7.13 is derived from clinical strain B128, isolated from a patient with a duodenal ulcer. This study reveals genes associated with the virulence of the strain.

## GENOME ANNOUNCEMENT

*Helicobacter pylori* is a Gram-negative microaerophilic spiral bacterium. *H. pylori* is etiologically associated with chronic gastritis, peptic ulcer disease, and gastric cancer in humans. Several virulence factors play an important role in the pathogenesis of *H. pylori*-associated disease, including cytotoxin-associated gene A (*cagA*), vacuolating cytotoxin A (*vacA*), and induction by contact with epithelium gene A (*iceA*) (1). Gerbil-adapted strain *H. pylori* 7.13 is a *cagA*^+^ strain and translocates the CagA protein into epithelial cells, and this strain causes apoptosis and DNA damage in mouse, gerbil, and human gastric epithelial cells (2, 3).

The genome of *H. pylori* strain 7.13 was sequenced using Illumina NextSeq 500 at Xcelris Labs. Ltd. The genome was sequenced with an average sequencing coverage of ~400×. The raw reads were quality filtered using Trimmomatic-0.30, and *de novo* assembly was carried out using ABySS, which generated 27 scaffolds (4, 5). The total assembly size obtained was 1.7 Mb, with a G+C content of 38%*.*

The genome was annotated using the Rapid Annotations using Subsystems Technology (RAST) server (6). A total of 1,707 potential coding sequences (CDSs) were identified, which included 277 hypothetical genes and 44 RNA genes. Of the 44 RNA genes, 36 are predicted to code for tRNA and eight for rRNA, including four large subunits and four small subunits of the ribosome. The sequence of this strain indicates the presence of a *cag* pathogenicity island (*cag* PAI) and the *vacA* and *iceA* genes.

### Nucleotide sequence accession numbers.

The genome sequence of *H. pylori* strain 7.13 was deposited in DDBJ/EMBL/GenBank with the accession no. LAQK00000000. The version of the strain described in this paper is the first version, LAQK01000000.
